# Higher Controlling Nutritional Status (CONUT) score indicates increased risk of sarcopenia in elderly hospitalized patients: a single institution study in China

**DOI:** 10.3389/fnut.2025.1669225

**Published:** 2025-11-06

**Authors:** Leying Sun, Yilin Yang, Ruiyi Yan, Bingqing Xu, Kaiyu Zhang, Wenyu Zhu, Xiaoyi Lian, Yihui Xu, Lei Liu, Xiuming Gao, Zhengli Guo, Mingqin Zhou

**Affiliations:** 1 Department of Rehabilitation Medicine, Affiliated Hospital of Xuzhou Medical University, Xuzhou, Jiangsu, China; 2 The Second Clinical School of Xuzhou Medical University, Xuzhou, Jiangsu, China; 3 School of Medicine of Southeast University, Nanjing, Jiangsu, China; 4 Eight-Year Medical Doctor Program, Peking Union Medical College Hospital, Chinese Academy of Medical Sciences and Peking Union Medical College, Beijing, China; 5 Department of Gerontology, Affiliated Kunshan Hospital of Jiangsu University, Kunshan, Jiangsu, China; 6 Department of Gerontology, Suzhou Municipal Hospital, Suzhou, Jiangsu, China; 7 Department of Critical Care Medicine, Cancer Hospital of Shantou University Medical College, Shantou, Guangdong, China

**Keywords:** Controlling Nutritional Status (CONUT), sarcopenia, aging, risk factor, nutritional status

## Abstract

**Background:**

The Controlled Nutritional Status (CONUT) metric has demonstrated effectiveness as a prognostic indicator for acute and chronic diseases in addition to other wasting conditions. However, its association with sarcopenia in elderly hospitalized patients remains insufficiently explored. Our study objectives included the assessment of the potential of CONUT score to predict sarcopenia onset.

**Methods:**

Our study was a single center retrospective cohort study. Patients from the Department of Geriatrics of the First People’s Hospital of Kunshan were recruited for this study. Multiple indicators related to nutrition and sarcopenia, including CONUT, Prognostic Nutritional Index (PNI), triglyceride–total cholesterol–body weight index (TCBI), Geriatric Nutritional Risk Index (GNRI), and handgrip strength (HGS). Spearman’s and Pearson’s correlation were calculated to assess the associations between nutritional indices and sarcopenia-related indicators. The demographic characteristics, physical examination findings and laboratory parameters were included in univariate logistic regression. Based on the results of univariate logistic regression and theoretical analysis, variables were selected for multivariate logistic regression in order to identify risk factors for sarcopenia.

**Results:**

A total of 236 elderly hospitalized patients were included. Malnutrition was prevalent in patients with sarcopenia. The optimal CONUT cut-off values were defined as >4 for males and >3 for females, dividing patients into high CONUT (*n* = 140, 59.32%) and low CONUT (*n* = 96, 40.58%) groups. Patients in the high CONUT group had lower levels of albumin, prealbumin, hemoglobin, and total lymphocyte count. Multivariate logistic regression analysis showed that a high CONUT score was an independent risk factor for sarcopenia (*OR:1.814, 95% CI: 1.019–3.255, p = 0.044*). *Male sex and low iron level were also demonstrated to be associated with sarcopenia.*

**Conclusion:**

CONUT score is an independent risk factor for sarcopenia and may serve as a practical indicator for sarcopenia risk screening in elderly hospitalized patients.

## 1 Introduction

Sarcopenia is a progressive, widespread skeletal muscle disorder (1–4), affecting approximately 10% of adults aged ≥60 years globally and predicted to impact over 500 million people worldwide by 2050 (4). The severe consequences of sarcopenia adversely affect the quality of life of older individuals, place greater demands on families, and pose a significant public health challenge. The current management of sarcopenia focuses primarily on resistance exercise and nutritional interventions (5, 6). Given the absence of specific pharmacotherapies for sarcopenia, identifying its modifiable risk factors is critical for developing targeted preventive strategies. Sarcopenia is positively associated with various disorders, including diabetes and its complications, osteoporosis, anorexia nervosa, cardiovascular disease, respiratory disease, arthritis, and metabolic disorders (7–11). However, current interventions have been ineffective, highlighting the need to explore new, easy-to-intervene risk factors.

Maintaining the nutritional status is essential for good health and skeletal muscle maintenance. Nutritional status monitoring is an effective approach for disease prediction (12, 13). Previous research has demonstrated a direct relationship between insufficient nutrition and the development of sarcopenia (6, 14). The symptoms of sarcopenia can be improved by supplementation with whey protein, essential amino acids, and vitamin D (15). Leucine-rich proteins, which possess specific synthetic functions, can fundamentally conserve muscles and impede the progression of skeletal sarcopenia (16–18). The European Society of Parenteral and Enteral Nutrition (ESPEN) also argues that moderate protein consumption can prevent illnesses.

Regarding the diagnosis of malnutrition, there remains no validated gold standard, and existing methods are generally inadequate for routine clinical practice (19, 20). Accordingly, we evaluated the association between the Controlling Nutritional Status (CONUT) score, a simple and readily calculable clinical metric, and sarcopenia. The CONUT score is constructed using three biomarkers: serum albumin, total lymphocyte count, and total cholesterol (TC) levels, and it serves as a practical indicator of a patient’s nutritional and immune status (21). Specifically, albumin is a well-recognized marker of systemic protein reserves, total lymphocyte count reflects the integrity of cellular immune function. Additionally, TC can indirectly reflect energy metabolism status, as reduced TC levels are often associated with insufficient energy intake or increased energy expenditure in elderly populations (21). The CONUT score correlates with the functional status reflected by these biomarkers: a higher score, accompanied by lower levels of the three nutrients, is associated with a greater decline in protein reserve, immune function, and energy metabolism. To date, this score has shown promising predictive value across various clinical contexts.

Compared to other nutritional indicators such as PNI and GNRI, CONUT offers unique advantages. It comprehensively integrates three key dimensions including protein status, immune function, and energy metabolism, thus providing a more holistic assessment of nutritional status, while also being simpler to calculate. Initially developed and validated in surgical and oncology departments, the CONUT score was designed to predict acute deterioration during hospitalization (21–24). However, recent analyses have demonstrated its significant prognostic relevance in diverse clinical conditions, including chronic diseases, cancer, and cardiac disorders (19, 25–27). Despite the well-established predictive value of the CONUT score for numerous conditions, research reports on its association with sarcopenia remain relatively limited.

The purpose of this study was therefore to examine the association between nutritional indicators, such as CONUT score, and sarcopenia development. Additionally, this study aimed to explore the risk factors for the development of sarcopenia, which would provide support for effective clinical prediction of sarcopenia.

## 2 Materials and methods

### 2.1 Participants

This is a single-centre, retrospective cohort study. A total of 236 patients from the Department of Geriatrics at First People’s Hospital of Kunshan were retrospectively recruited for this study. The inclusion criteria were as follows: (1) age over 60 years and (2) complete information about the clinicopathological diagnosis. The exclusion criteria included (1) age less than 60 years; (2) unclear diagnosis; (3) lack of information about the clinicopathological diagnosis; (4) recent or current use of drugs that cause muscle damage, such as statins; (5) limb impairment that prevents physical tests, such as grip strength, four-metre step test, 5-time chair stand test, balance test, and others; and (6) refusal or legal incapacity to provide informed consent. Patients with missing data were excluded, the overall missing data rate was <5%.

### 2.2 Assessment of sarcopenia

The Asian Working Group for Sarcopenia (AWGS) criteria proposed in 2019 (5) were used to diagnose sarcopenia in this study. The diagnostic criteria for sarcopenia in these guidelines include decreased grip strength and/or usual stride speed, as well as decreased skeletal muscle mass. Grip strength was measured using a CAMRY EH101 electronic hand dynamometer on the dominant hand. Patients with a usual step speed of less than 1.0 m/s on a 6-metre walk test were classified as having a positive result (5). Skeletal muscle mass (ASM) was measured by DXA scanning, following the established criteria. Skeletal muscle mass was measured using the Relative Skeletal Muscle Mass Index (RSMI) (ASM/height^2^), which is also a prerequisite for the diagnosis of sarcopenia. Measurements were taken from stable patients without severe inflammation.

### 2.3 Data collection

Demographic data, physical examination findings, medical history, and serum laboratory test results were retrospectively collected. The demographic data included age, sex, and body mass index (BMI). Physical examination findings included relative skeletal muscle mass index (RSMI), handgrip strength (HGS), a four-metre step test, a 5-time chair stand test, and balance test. Technical term abbreviations such as RSMI and HGS were explained upon first use. The medical history included hypertension, diabetes mellitus, osteoporosis, COPD, atherosclerosis, cancer, neuromuscular disorders, and renal failure. Additionally, serum laboratory test results were collected, comprising of serum albumin concentration (ALB), prealbumin (PA), hemoglobin (Hb), transferrin (TRF), serum total cholesterol (TC), triglyceride (TG), total peripheral lymphocyte (TLC), fasting blood glucose (FBG), iron (Fe), zinc (Zn), and calcium (Ca). A manual screening evaluation was conducted to collect clinical data.

For the four-metre step test, a speed of 1 m/s or lower was deemed positive, whereas anything higher was classified as negative. During the balance trials, patients were asked to stand in three different positions: feet side by side, one heel at the midpoint of the other foot, and feet in a line, heel to toe. A positive result was determined if balance could not be maintained for more than 10 s in any of the three specified positions. For the 5-time chair stand test, patients were required to stand up from a chair with a standard height of 43 cm and cross their arms over their chest 5 times. A total time of 12 s or more was considered a positive result. These tests are based on the AWGS Consensus on the Diagnosis and Treatment of Sarcopenia, which was updated in 2019 (5).

The PNI is computed by adding 10 times the ALB in grams per litre (g/L) to 0.005 times the total lymphocyte count per cubic millimeter (/mm^3^) (28). The triglyceride–TCBI was determined by multiplying the values for serum total cholesterol (TC) in milligrams per deciliter (mg/dL), triglyceride (TG) in mg/dL, and body weight in kilograms (/kg), and then dividing the product by 1,000 (29). The Geriatric Nutritional Risk Index (GNRI) was calculated as follows: GNRI = 1.487 × ALB (g/L) + 41.7 × weight/ideal weight (kg). The ideal body weight was calculated as: ideal bodyweight = 22 × square of height (m) (30).

### 2.4 Controlling nutritional status score cut-off value

The CONUT score is commonly used to evaluate nutritional status. The CONUT score was determined by combining ALB, TLC, and TC. These three indicators were combined to derive the corresponding CONUT scores, as illustrated in Supplementary Table 1. A score of less than 2 on the CONUT scale indicates normal nutritional status, while scores ranging between 2 and 4 indicate mild malnutrition. Scores between 5 and 8 indicate moderate malnutrition, and a score of 9 or above indicate severe malnutrition.

### 2.5 Statistical analysis

SPSS version 26.0 (IBM Corp., Armonk, NY, USA) was used for statistical analyses. Categorical variables are presented as numbers and percentages, and continuous variables are presented as medians, first quartiles, and third quartiles. Cut-off values commonly used in clinical practice were employed to convert continuous variables, such as BMI, ALB, Hb, and FBG, into categorical variables. The optimal cut-off values for PNI, TCBI, and GNRI were calculated using Receiver Operating Characteristic (ROC) analysis. As the study population was elderly, and some indicators, such as TLC, were different from those of the normal population, the median was used as the cut-off value. Considering the differences in CONUT scores between males and females, the cut-off values were divided based on the median CONUT scores for males and females, respectively. This approach was chosen to account for potential physiological differences in nutritional and immune markers between sexes that could influence baseline CONUT scores. Hence, patients were categorized into two groups based on the cut-off value: those with high CONUT scores and those with low CONUT scores.

The Mann–Whitney U test was used to examine differences between groups for continuous variables, while for categorical variables, either Pearson’s or Fisher’s exact chi-square test was employed. Spearman’s rank correlation analysis and Pearson’s correlation analysis were performed to examine the correlations between various nutritional scores, including BMI, CONUT, PNI, TCBI, and GNRI in patients with and without sarcopenia. Logistic regression was used to study the risk factors for sarcopenia development and to predict the risk of developing sarcopenia based on nutritional scores. First, univariate analysis of risk factors was conducted. Indicators with a *p*-value < 0.05 were included in the multivariate logistic regression to estimate the associated odds ratios (OR) and 95% confidence intervals (CI). Two-sided tests were used for all evaluations, and statistical significance was set at *p* < 0.05.

## 3 Results

### 3.1 Patient baseline characteristics

A total of 236 patients were enrolled in this study, and their baseline information is shown in Table 1. The median patient age was 72 years. A total of 148 patients were female (62.71%) and 88 patients were male (37.29%). The median BMI (kg/m^2^), RSMI, and HGS (kg) were 23.6 kg/m^2^, 5.77 and 20.00 kg, respectively. All the above indices were higher in non-sarcopenic patients than in sarcopenic patients and were statistically significant (all *p* < 0.05). The number of positive results in the four-metre step test, sit-to-up test, and balance test were 107 (45.34%), 142 (60.17%), and 19 (8.05%), respectively. Twenty-three (9.75%) of the patients had or have had cancer, which was significantly higher in sarcopenia patients than in non-sarcopenia patients (13.91% vs. 5.79%, *p* = 0.035). The percentage of neuropsychiatric disorders was also higher in patients with sarcopenia than in the group without sarcopenia (14.78% vs. 6.61%, *p* = 0.041). Among laboratory parameters, non-sarcopenic patients had significantly higher median levels of albumin (ALB), prealbumin (PA), hemoglobin (Hb), transferrin (TRF), total cholesterol (TC), triglycerides (TG), total lymphocyte count (TLC), fasting blood glucose (FBG), and Fe (μmol/L) than sarcopenic patients (all *p* < 0.05). Additionally, triglyceride levels in patients with sarcopenia were only three-quarters of those in patients without sarcopenia. Serum prealbumin and hemoglobin levels in both groups were slightly above the lower bounds of normal values, whereas iron levels were lower than the standard range for adults.

**Table 1:** General characteristics of participants with sarcopenia.

Characteristic	Total	Sarcopenia (*n* = 115)	Non sarcopenia (*n* = 121)	*p*
**Age (years)**	72 (67–72)	75 (70–80)	69 (65–74.5)	**<0.001**
**Sex**				**0.001**
Female	148 (62.71)	60 (52.17)	88 (72.72)	
Male	88 (37.29)	55 (47.83)	33 (27.28)	
**BMI (kg/m** ^**2**^ **)**	23.6 (20.93–25.88)	21.60 (19.50–24.10)	25.00 (23.14–27.00)	**<0.001**
**RSMI**	5.77 (5.25–6.48)	5.32 (4.80–5.87)	6.29 (5.70–7.11)	**<0.001**
**HGS (kg)**	20.00 (15.03–25.58)	19.70 (14.40–25.40)	20.45 (15.55–28.70)	**0.029**
**Four-metre step test *n* (%)**				0.125
Negative	129 (54.66)	57 (49.57)	72 (59.50)	
Positive	107 (45.34)	58 (50.43)	49 (40.50)	
**5-time chair stand test *n* (%)**				**0.009**
Negative	94 (39.83)	36 (31.30)	58 (47.93)	
Positive	142 (60.17)	79 (68.70)	63 (52.07)	
**Balance test *n* (%)**				0.073
Negative	217 (91.95)	102 (88.70)	115 (95.04)	
Positive	19 (8.05)	13 (11.30)	6 (4.96)	
**Hypertension *n* (%)**	138 (58.47)	64 (55.65)	74 (61.16)	0.391
**Diabetes *n* (%)**	62 (26.27)	30 (26.09)	32 (26.45)	0.950
**Osteoporosis *n* (%)**	127 (53.81)	62 (53.91)	65 (53.72)	0.976
**COPD *n* (%)**	41 (17.37)	25 (21.74)	16 (13.22)	0.084
**Atherosclerosis *n* (%)**	116 (49.15)	54 (46.96)	62 (51.24)	0.511
**Cancer *n* (%)**	23 (9.75)	16 (13.91)	7 (5.79)	**0.035**
**Neuromuscular aspects *n* (%)**	25 (10.59)	17 (14.78)	8 (6.61)	**0.041**
**Renal failure *n* (%)**	4 (1.69)	3 (2.61)	1 (0.83)	0.578
Laboratory data				
ALB (g/L)	39.25 (36.63–41.70)	38.40 (36.10–40.40)	39.70 (37.50–42.50)	**0.001**
PA (mg/L)	223.00 (184.25–253.75)	215.00 (171.00–241.00)	224.00 (196.50–267.00)	**0.013**
Hb (mg/L)	126.95 (116.13–134.83)	125.00 (111.00–133.00)	129.20 (121.00–136.55)	**0.004**
TRF (g/L)	2.05 (1.84–2.23)	2.00 (1.74–2.18)	2.05 (1.91–2.35)	**0.002**
TC (mmol/L)	4.13 (3.59–4.85)	4.03 (3.52–4.64)	4.39 (3.68–4.99)	**0.034**
TG (mmol/L)	1.07 (0.80–1.55)	0.93 (0.74–1.32)	1.21 (0.89–1.67)	**0.001**
TLC (mmol/L)	1.31 (1.00–1.70)	1.20 (0.90–1.52)	1.41 (1.12–1.87)	**<0.001**
FBG (mmol/L)	5.14 (4.70–5.91)	5.00 (4.59–5.76)	5.27 (4.84–5.99)	**0.007**
Fe (μmol/L)	7.80 (7.62–8.09)	7.73 (7.59–8.01)	7.88 (7.65–8.16)	**0.013**
Zn (μmol/L)	85.77 (79.53–91.54)	85.62 (78.26–90.82)	85.77 (79.58–92.39)	0.215
Ca (mmol/L)	1.57 (1.49–1.63)	1.58 (1.49–1.67)	1.56 (1.49–1.62)	0.354

Values are presented as number (%) or median (range). BMI, body mass index; RSMI, relative skeletal muscle mass index; HGS, handgrip strength; COPD, chronic obstructive pulmonary disease; ALB, serum albumin concentration; PA, prealbumin; Hb, hemoglobin; TRF, transferrin; TC, serum total cholesterol; TG, triglyceride; TLC, total peripheral lymphocyte; FBG, fasting blood glucose; Fe, iron; Zn, zinc; Ca, calcium.

### 3.2 Differences in nutritional scores between sarcopenia and non-sarcopenia patients

The nutritional status of patients is commonly evaluated using various scores including BMI, CONUT, PNI, TCBI, and GNRI. Table 2 shows the differences in nutritional scores between patients with and without sarcopenia. The results indicated that sarcopenia patients had higher CONUT scores compared to non-sarcopenia patients (5 vs. 4, *p* < 0.001). Comparing the BMI of the two groups, it was found that the majority of sarcopenic patients had normal levels of adiposity, while a proportion of non-sarcopenic patients were classified as overweight. In contrast, PNI, TCBI, and GNRI were significantly lower in sarcopenic patients than in non-sarcopenic patients (all *p* < 0.05), consistent with poorer nutritional status in the sarcopenia group.

**Table 2:** Differences in nutritional scores between sarcopenia and non-sarcopenia patients.

Nutritional score	Sarcopenia	Non sarcopenia	*p*
BMI	21.60 (19.50–24.10)	25.00 (23.14–27.00)	**<0.001**
CONUT	5 (4–5)	4 (3–5)	**<0.001**
PNI	44.30 (41.05–48.10)	47.65 (44.50–51.15)	**<0.001**
TCBI	728.13 (452.10–1121.83)	1086.25 (742.11–1662.01)	**<0.001**
GNRI	98.58 (91.59–104.49)	107.83 (100.41–113.12)	**<0.001**

BMI, body mass index; CONUT, controlling nutritional status score; PNI, prognostic nutritional index; TCBI, triglyceride–total cholesterol–body weight index; GNRI, geriatric nutritional risk index.

### 3.3 Association of nutrition score with sarcopenia assessment

The correlations between the nutritional scores and RSMI, HGS, four-metre step test, 5-time chair stand test and balance test in patients with and without sarcopenia are shown in Table 3; Supplementary Figure 5. The results showed a lack of significant correlation between most nutritional scores and the indices used to assess sarcopenia in both groups. The PNI did not correlate with any of the parameters in either group, however, correlations between BMI, CONUT, TCBI, GNRI, and some of the parameters were evident. In both groups, there was a significant correlation between BMI, GNRI, and RSMI; however, this correlation was higher in patients with sarcopenia than in non-sarcopenia patients.

**Table 3:** Correlations of nutrition score with sarcopenia assessment.

Nutritional score		RSMI	HGS	Four-metre step test	5-time chair stand test	Balance test
		*r*	*r*	*r*	*r*	*r*
		*p*	*p*	*p*	*p*	*p*
0	BMI	0.441	−0.054	−0.067	0.221	0.090
		**<0.001**	0.556	0.467	**0.015**	0.327
1		0.454	0.018	0.101	0.152	0.065
		**<0.001**	0.847	0.283	0.106	0.490
0	CONUT	−0.017	0.215	−0.082	−0.166	0.003
		0.855	**0.018**	0.373	0.068	0.970
1		0.126	0.116	−0.039	−0.076	0.118
		0.181	0.217	0.681	0.418	0.207
0	PNI	0.046	0.044	−0.054	0.018	−0.011
		0.615	0.629	0.556	0.843	0.901
1		0.044	−0.012	−0.065	0.103	−0.162
		0.643	0.898	0.489	0.276	0.084
0	TCBI	0.128	0.001	−0.060	−0.011	0.097
		0.162	0.990	0.512	0.906	0.290
1		0.185	−0.034	−0.053	0.226	−0.102
		**0.048**	0.722	0.571	**0.015**	0.279
0	GNRI	0.299	0.113	−0.157	0.010	0.104
		**0.001**	0.218	0.085	0.910	0.258
1		0.395	0.058	−0.015	0.150	−0.041
		**<0.001**	0.539	0.872	0.110	0.667

0 = Non-sarcopenia; 1 = Sarcopenia. Spearman’s *r* was used for correlations between non-normal distribution variables and Pearson’s *r* for correlations between normal distribution variables, and *p* < 0.05 was considered statistically significant. RSMI, relative skeletal muscle mass index; HGS, handgrip strength; BMI, body mass index; CONUT, controlling nutritional status score; PNI, prognostic nutritional index; TCBI, triglyceride–total cholesterol–body weight index; GNRI, geriatric nutritional risk index.

### 3.4 Relationship between CONUT and clinicopathological parameters

This study found a difference in CONUT scores between men and women. We calculated the median CONUT scores of 4 and 3 for the male and female groups, respectively, and used these as cut-off values. Based on these cut-off values, we divided the 236 patients into High CONUT and low CONUT score groups and summarized the clinical data of both groups in Table 4. A comparison of the nutritional scores of the PNI, TCBI, and GNRI between the two groups demonstrated higher values for all three indicators in the low CONUT group compared to the high CONUT score group (*p* < 0.05). This suggests that the nutritional status of patients in the high CONUT group was poorer than that of patients in the low CONUT group, which is consistent with the concept that increasing CONUT values indicating a worse nutritional status. Regarding serological testing, patients in the high CONUT score group exhibited lower levels of albumin, prealbumin, hemoglobin, and serum lymphocyte counts than those in the low CONUT score group (*p* < 0.05). This indicates that patients in the high CONUT score group may experience compromised protein synthesis and immunity, as well as a heightened likelihood of developing anemia.

**Table 4:** The relationship between the CONUT and clinical characteristics of patients with sarcopenia.

Characteristic	Total	High CONUT (*n* = 79)	Low CONUT (*n* = 36)	*p*
**Age (years)**	75 (70–80)	77 (71–81)	72.5 (68.25–78.75)	0.112
**Sex**				0.624
Female	60 (52.17)	40 (50.63)	20 (55.56)	
Male	55 (47.83)	39 (49.37)	16 (44.44)	
**BMI (kg/m** ^**2**^ **)**	21.60 (19.50–24.10)	21.60 (19.40–24.20)	22.10 (19.58–23.98)	0.916
**RSMI**	5.32 (4.80–5.87)	5.29 (4.83–5.92)	5.35 (4.76–5.87)	0.861
**HGS (kg)**	19.70 (14.40–25.40)	19.40 (14.05–23.50)	20.40 (15.03–22.75)	0.880
**Four-metre step test *n* (%)**				0.734
Negative	57 (49.57)	40 (50.63)	17 (47.22)	
Positive	58 (50.43)	39 (49.37)	19 (52.78)	
**5-time chair stand test *n* (%)**				0.582
Negative	36 (31.30)	26 (32.91)	10 (27.78)	
Positive	79 (68.70)	53 (67.09)	26 (72.22)	
**Balance test *n* (%)**				0.965
Negative	102 (88.70)	70 (88.61)	32 (88.89)	
Positive	13 (11.30)	9 (11.39)	4 (11.11)	
**Hypertension *n* (%)**	64 (55.65)	46 (58.23)	18 (50.00)	0.410
**Diabetes *n* (%)**	30 (26.09)	21 (26.58)	9 (25.00)	0.858
**Osteoporosis *n* (%)**	62 (53.91)	43 (54.43)	19 (52.78)	0.869
**COPD *n* (%)**	25 (21.74)	15 (18.99)	10 (27.78)	0.289
**Atherosclerosis *n* (%)**	54 (46.96)	36 (45.57)	18 (50.00)	0.659
**Cancer *n* (%)**	16 (13.91)	13 (16.46)	3 (8.33)	0.243
**Neuromuscular aspects *n* (%)**	17 (14.78)	13 (16.46)	4 (11.11)	0.454
**Renal failure *n* (%)**	3 (2.61)	1 (1.27)	2 (5.56)	0.230
Nutrition score				
PNI	44.30 (41.05–48.10)	42.80 (39.40–45.20)	48.75 (46.70–48.75)	**<0.001**
TCBI	728.13 (452.10–1121.83)	718.44 (436.97–1012.05)	770.27 (515.02–1733.72)	**0.043**
GNRI	98.58 (91.59–104.49)	96.65 (90.44–102.71)	100.15 (95.50–108.34)	**0.010**
Laboratory data				
ALB (g/L)	38.40 (36.10–40.40)	37.30 (34.90–39.80)	39.85 (37.20–42.40)	**<0.001**
PA (mg/L)	215.00 (171.00–241.00)	198.00 (161.00–230.00)	237.00 (204.25–283.50)	**<0.001**
Hb (mg/L)	125.00 (111.00–133.00)	121.00 (109.00–130.20)	129.00 (123.00–135.75)	**0.019**
TRF (g/L)	2.00 (1.74–2.18)	2.00 (1.73–2.18)	2.00 (1.76–2.18)	0.823
TC (mmol/L)	4.03 (3.52–4.64)	3.89(3.48–4.51)	4.34 (3.55–5.24)	0.070
TG (mmol/L)	0.93 (0.74–1.32)	0.92(0.71–1.23)	1.04 (0.74–1.88)	0.134
TLC (mmol/L)	1.20 (0.90–1.52)	1.01(0.84–1.24)	1.69 (1.50–2.05)	**<0.001**
FBG (mmol/L)	5.00 (4.59–5.76)	5.00 (4.59–5.67)	4.89 (4.55–5.90)	0.802
Fe (μmol/L)	7.73 (7.59–8.01)	7.68 (7.56–8.01)	7.75 (7.64–8.03)	0.431
Zn (μmol/L)	85.62 (78.26–90.82)	85.77 (78.12–91.03)	85.47 (80.95–90.29)	0.845
Ca (mmol/L)	1.58 (1.49–1.67)	1.58 (1.49–1.69)	1.56 (1.47–1.67)	0.616

Values are presented as number (%) or median (range). BMI, body mass index; RSMI, relative skeletal muscle mass index; HGS, handgrip strength; COPD, chronic obstructive pulmonary disease; CONUT, controlling nutritional status score; PNI, prognostic nutritional index; TCBI, triglyceride–total cholesterol–body weight index; GNRI, geriatric nutritional risk index; ALB, serum albumin concentration; PA, prealbumin; Hb, hemoglobin; TRF, transferrin; TC, serum total cholesterol; TG, triglyceride; TLC, total peripheral lymphocyte; FBG, fasting blood glucose; Fe, iron; Zn, zinc; Ca, calcium.

### 3.5 Factors predicting sarcopenia

**Figure 1:**
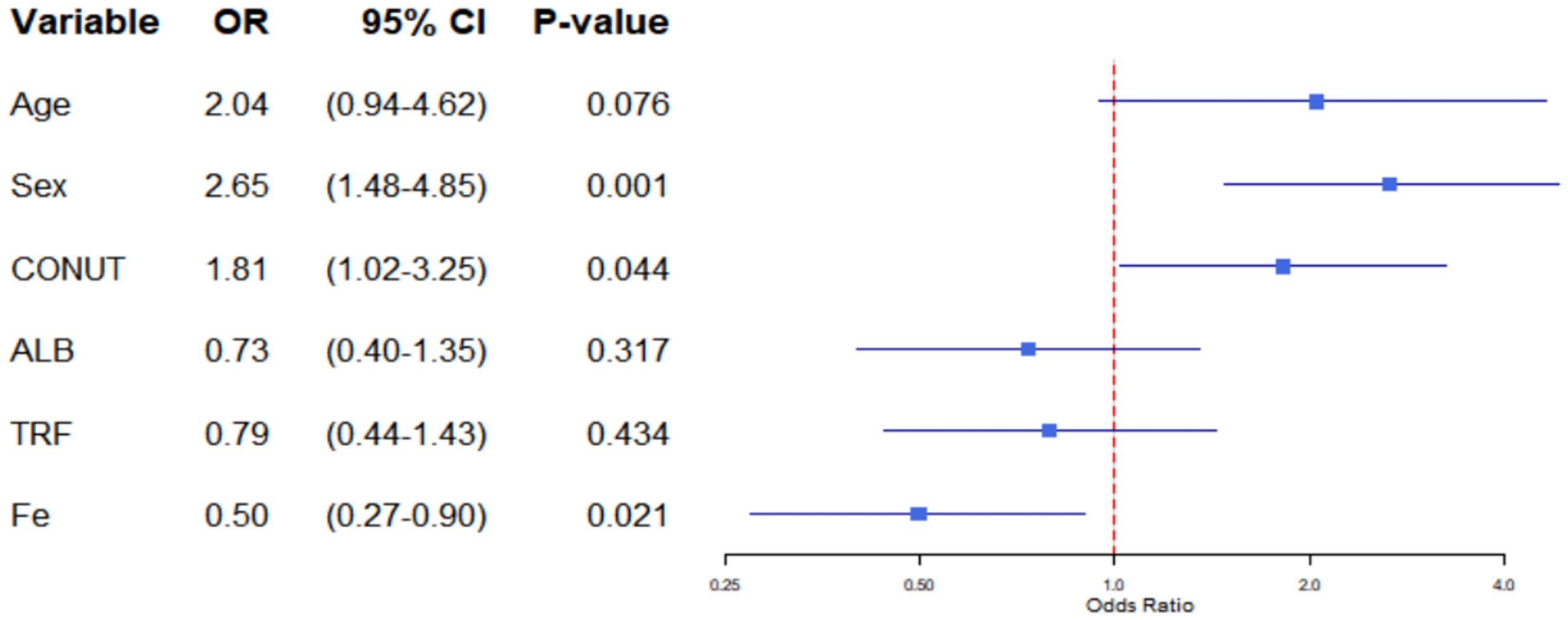
Forest plot for multivariate Logistics analysis.

The findings from the univariate and multivariate regression analyses of the 236 patients are displayed in Table 5; Figure 1. In terms of demographics, patients who were male, older (age greater than 65 years), and had a low BMI (BMI ≤ 24 kg/m^2^) were more likely to develop sarcopenia. On physical examination, low RSMI (<7.0 kg/m^2^ in male and <5.6 kg/m^2^ in female), low HGS (<26 kg in men and <18 kg in women), and a positive 5-time chair stand test were positively associated with sarcopenia. However, the results of the balance test and the four-metre step test showed no significant correlation with sarcopenia. Patients with a history of cancer or neuromuscular disease are also more likely to develop sarcopenia. Unexpectedly, no correlation was observed between diabetes, osteoporosis, and sarcopenia. This result differed from that of Izzo et al. (30). In terms of diet, all four commonly used nutritional scores included in this study were associated with sarcopenia. CONUT male > 4, female > 3, PNI ≤ 42.93, TCBI ≤ 865 and GNRI ≤ 98 were found to be risk factors for the development of sarcopenia. Additionally, in terms of laboratory tests, higher ALB, Hb, TRF and TLC were proven to be protective factors for the development of sarcopenia. Similar to the past medical history, fasting blood glucose was not significantly correlated with sarcopenia. Among the trace elements, Fe has been shown to be a risk factor for sarcopenia. However, the Ca and Zn levels were not correlated with sarcopenia.

**Table 5:** Univariate and multivariate logistic regression analysis in 236 patients.

Characteristic	Univariate analysis			Multivariate analysis		
	OR	95% CI	*p*	OR	95% CI	*p*
Age (>65 year vs. ≤65 year)	3.086	1.500–6.350	**0.002**	2.043	0.944–4.620	0.076
Sex (male vs. female)	2.444	1.421–4.204	**0.001**	2.652	1.479–4.848	**0.001**
BMI (>24 kg/m^2^ vs. ≤24 kg/m^2^)	0.186	0.106–0.326	**<0.001**	–	–	–
RSMI (male < 7.0 kg/m^2^ female < 5.6 kg/m^2^ vs. male ≥ 7.0 kg/m^2^ female ≥ 5.6 kg/m^2^)	0.013	0.004–0.044	**<0.001**	–	–	–
HGS (male > 26 kg female > 18 kg vs. male ≤ 26 kg female ≤ 18 kg)	0.245	0.142–0.423	**<0.001**	–	–	–
Four-metre step test (positive vs. negative)	1.495	0.893–2.503	0.126	–	–	–
5-time chair stand test (positive vs. negative)	2.020	1.187–3.438	**0.010**	–	–	–
Balance test (positive vs. negative)	2.443	0.896–6.663	0.081	–	–	–
Hypertension (yes vs. no)	0.797	0.474–1.339	0.391	–	–	–
Diabetes (yes vs. no)	0.982	0.550–1.753	0.950	–	–	–
Osteoporosis (yes vs. no)	1.008	0.604–1.682	0.976	–	–	–
COPD (yes vs. no)	1.823	0.916–3.626	0.087	–	–	–
Atherosclerosis (yes vs. no)	0.842	0.505–1.404	0.511	–	–	–
Cancer (yes vs. no)	2.632	1.040–6.658	**0.041**	–	–	–
Neuromuscular aspects (yes vs. no)	2.450	1.013–5.924	**0.047**	–	–	–
Renal failure (yes vs. no)	3.214	0.330–31.355	0.315	–	–	–
Nutrition score						
CONUT (male > 4 female > 3 vs. male ≤ 4 female ≤ 3)	2.158	1.269–3.672	**0.005**	1.814	1.019–3.255	**0.044**
PNI (>42.93 vs. ≤42.93)	0.372	0.205–0.676	**0.001**	–	–	–
TCBI (>865 vs. ≤865)	0.283	0.165–0.485	**<0.001**	–	–	–
GNRI (>98 vs. ≤98)	0.191	0.103–0.354	**<0.001**	–	–	–
Laboratory data						
ALB (>38 g/dL vs. ≤38 g/dL)	0.498	0.292–0.848	**0.010**	0.734	0.400–1.347	0.317
PA (>280 mg/L vs. ≤280 mg/L)	0.758	0.367–1.563	0.452	–	–	–
Hb (>120 mg/L vs. ≤120 mg/L)	0.473	0.270–0.828	**0.009**	–	–	–
TRF (>2 g/L vs. ≤2 g/L)	0.524	0.311–0.882	**0.015**	0.790	0.439–1.429	0.434
TC (>3.6 mmol/L vs. ≤3.6 mmol/L)	0.781	0.434–1.405	0.409	–	–	–
TG (>0.56 mmol/L vs. ≤0.56 mmol/L)	0.773	0.233–0.703	0.567	–	–	–
TLC (>1.3 mmol/L vs. ≤1.3 mmol/L)	0.542	0.323–0.908	**0.020**	–	–	–
FBG (>6.1 mmol/L vs. ≤6.1 mmol/L)	0.699	0.368–1.328	0.274	–	–	–
Fe (>7.67 μmol/L vs. ≤7.67 μmol/L)	0.460	0.269–0.787	**0.005**	0.498	0.273–0.895	**0.021**
Zn (>85.41 μmol/L vs. ≤85.41 μmol/L)	1.011	0.605–1.690	0.968	–	–	–
Ca (>1.57 mmol/L vs. ≤1.57 mmol/L)	1.105	0.663–1.842	0.701	–	–	–

Values are presented as number (%) or median (range). BMI, body mass index; RSMI, relative skeletal muscle mass index; HGS, handgrip strength; COPD, chronic obstructive pulmonary disease; CONUT, controlling nutritional status score; PNI, prognostic nutritional index; TCBI, triglyceride–total cholesterol–body weight index; GNRI, geriatric nutritional risk index; ALB, serum albumin concentration; PA, prealbumin; Hb, hemoglobin; TRF, transferrin; TC, serum total cholesterol; TG, triglyceride; TLC, total peripheral lymphocyte; FBG, fasting blood glucose; Fe, iron; Zn, zinc; Ca, calcium. The bolded parts 5 indicate that the *p*-value is less than 0.05.

Variables for multivariate logistic regression were selected by combining the results of univariate logistic regression with theoretical analysis to eliminate confounding factors (Table 5). The multivariate analysis showed that male sex (OR = 2.652, 95% CI: 1.479–4.848, *p* = 0.001), CONUT male > 4, female > 3 (OR = 1.814; 95% CI: 1.019–3.255; *p* = 0.044) and Fe ≤ 7.67 mol/L (OR = 0.498; 95% CI: 0.273–0.895; *p* = 0.021) were independent factors for the development of sarcopenia.

## 4 Discussion

The risk factors for sarcopenia are currently being investigated. As a result, the effective prediction and prevention of sarcopenia remain a substantial challenge, primarily in the elderly population. This study evaluated the association between novel nutritional indicators and sarcopenia. Our results showed that sarcopenic patients differed from non-sarcopenic patients in terms of BMI and multiple nutritional indices including CONUT, PNI, TCBI, and GNRI. Among these, the CONUT was identified as an independent risk factor for sarcopenia. However, its ability to predict sarcopenia requires further validation (Supplementary Figures 2–4).

Our study demonstrated that a high CONUT score is associated with an increased risk of sarcopenia, though the precise mechanisms underlying this association remain to be fully elucidated. Serum albumin, a key marker of systemic protein reserve, is directly linked to sarcopenia through two pathways supported by sarcopenia-specific observations. First, reduced albumin levels indicate insufficient dietary protein intake or impaired protein utilization—both well-documented risk factors for sarcopenia. A prospective study in community-dwelling older adults showed that hypoalbuminemia (serum albumin <3.8 g/dL) was associated with a 2.3-fold higher risk of incident sarcopenia, likely via limiting muscle protein synthesis (31). Second, hypoalbuminemia disrupts muscle microenvironment integrity by reducing intravascular colloid osmotic pressure, leading to interstitial edema. It might associate with impaired muscle contractility via increased tissue stiffness (32). Also, in patients with sarcopenia, reduced albumin levels and upregulated expression of inflammatory factors further diminish the synthesis of multiple proteins involved in amino acid conservation (33, 34). Notably, these links still require further validation in interventional trials.

Secondly, reduced total lymphocyte count contributes to sarcopenia via inflammatory dysregulation, with direct evidence from sarcopenia cohorts (5, 33, 35, 36). Mechanistically, lymphocyte depletion impairs regulatory T-cell function, which normally restricts excessive pro-inflammatory cytokine release. Previous studies have demonstrated potential causal correlation between IL-10, IP-10, M-CSF, Neutrophil-to-lymphocyte ratio (NLR) and sarcopenia-related traits (37, 38). Thus, reduced lymphocyte counts typically indicate impaired muscle regenerative potential.

Finally, decreased total cholesterol levels reflect caloric depletion and may hinder the synthesis of steroid hormones (39–41). Epidemiological evidence supporting this association comes from a cross-sectional study of 303 adults aged ≥60 years, which demonstrated that serum concentrations of TC, TG, and LDL were significantly lower in the sarcopenia group (*p* < 0.01) (42). The potential mechanism linking low TC to sarcopenia lies in cholesterol’s role as an essential precursor for testosterone and vitamin D biosynthesis (43–45). Both hormones play non-redundant roles in maintaining skeletal muscle homeostasis: testosterone supports muscle protein synthesis by activating the mTOR signaling pathway, while vitamin D regulates calcium handling in muscle fibers and preserves neuromuscular function.

These findings demonstrate that malnutrition is a potential pathogenic mechanism of sarcopenia. According to Papadopoulou et al. (6), protein and vitamin supplementation is essential for disease prevention. Importantly, leucine-rich proteins play vital roles. The reduction in protein degradation caused by lowering the ubiquitin pathway can be attributed to HMB, a leucine metabolite. This process yields the substrates required for cell membrane repair (46). Furthermore, a prudent dietary pattern may be useful in avoiding sarcopenia. A dietary plan that offers antioxidants may have a constructive impact on muscle sustenance (6). Therefore, we propose the prevention and resolution of sarcopenia from a nutritional perspective with a focus on precision.

Notably, when compared with other frequently employed nutritional indicators, CONUT either demonstrates advantages or exhibits no disadvantages. Hao et al. (47) have found that GNRI is a reliable predictor of sarcopenia in American adults aged 45 and above, but its calculation relies on ideal body weight, which may differ from the actual situation. This dependency makes GNRI vulnerable to confounding in populations with abnormal weight, as obesity and sarcopenia often coexist in older adults, which may obscure the true association between nutrition and sarcopenia. By contrast, the CONUT score is not directly dependent on body weight (unlike BMI, GNRI, and TCBI), thereby avoiding confounding by obesity or weight abnormalities. For example, in our cohort, sarcopenic patients had a significantly lower median BMI (21.60 kg/m^2^) than non-sarcopenic patients (25.00 kg/m^2^), but CONUT still independently predicted sarcopenia after adjusting for BMI, confirming its robustness to weight differences. For the PNI, Cheng et al. (48) reported that a higher PNI was associated with a lower incidence of sarcopenia in community-dwelling older adults, but PNI only integrates serum albumin and total lymphocyte count, lacking an indicator for energy metabolism.

In addition, the CONUT score is easy to calculate from a comprehensive blood count, making it an appropriate follow-up test. The preferred CONUT cut-off value differs among various studies (21–24). For gastric cancer, the optimum cut-off is 4 (21), whereas for esophageal cancer, it is 6 (22). However, Dalmiglio et al. (49) chose 3 as the optimal cut-off for predicting the prognosis of patients with advanced thyroid cancer treated with TKI. For patient grouping in this study, we considered a score of 4 for men and 3 for women as the optimal threshold. However, further validation is required to confirm whether this sex-specific CONUT cut-off is applicable to other sarcopenia populations.

Our study did not detect a significant association among diabetes, osteoporosis, and sarcopenia. The underlying reason might be that the elderly hospitalized patients included in our study may be afflicted with multiple acute or chronic conditions. These comorbidities have the potential to obscure the independent impact of diabetes or osteoporosis on sarcopenia. It was further demonstrated that three indicators (age, albumin and transferrin) exhibited statistical significance in univariate logistic regression. However, upon inclusion in multivariate regression, these indicators failed to demonstrate comparable results. Consequently, a multicollinearity analysis was conducted among the variables incorporated into the multivariate regression (Supplementary Table 2). The findings indicated the absence of multicollinearity among the variables, with any observed multicollinearity falling within acceptable limits. It is hypothesised that these three variables may exert confounding effects or mediate actions within the model. However, exploring causal relationships between variables requires a more rigorous experimental design.

However, our study has certain limitations. Firstly, as a single centre retrospective study, the present research is subject to selection bias arising from single centre patient recruitment and information bias stemming from retrospective data collection. This may have consequences for the accuracy and generalizability of our findings. In order to validate the findings in future, it is essential that larger-scale, multicenter prospective studies are conducted. Second, this study focused on the correlation between sarcopenia and nutritional scores (e.g., the CONUT score) in a specific population of older Chinese adults. Nevertheless, as distinct dietary habits and lifestyle patterns across various regions are prominent factors contributing to this disorder, research based on populations from other areas is crucial before generalizing the results of our study.

In conclusion, this study demonstrates that the CONUT score is associated with sarcopenia in elderly hospitalized patients. Additionally, CONUT may serve as a promising indicator for sarcopenia risk assessment. However, additional prospective, multicenter studies are needed to validate its predictive value across diverse populations.

## Data Availability

The datasets presented in this article are not readily available because data privacy policy at our facility, but are available from the corresponding author on reasonable request. Requests to access the datasets should be directed to Zhengli Guo, 350834416@qq.com.
