# Supplementary material for: Higher Controlling Nutritional Status (CONUT) score indicates increased risk of sarcopenia in elderly hospitalized patients: a single institution study in China
Source: Front Nutr. 2025 Nov 6;12:1669225. doi: 10.3389/fnut.2025.1669225 (PMC12631264; doi:10.3389/fnut.2025.1669225)
Supplement: Supplementary file 1 [file Supplementary_file_1.docx]

**Supplementary Materials**

Supplement Table 1. Definition of CONUT score

|  | Quantity Contained | Score |
| --- | --- | --- |
| ALB (g/L) | ≥35.00 | 0 |
|  | 30.00-34.90 | 2 |
|  | 25.00-29.90 | 4 |
|  | ≤24.90 | 6 |
| TLC(×10^9^/L) | ≥1.60 | 0 |
|  | 1.20-1.59 | 1 |
|  | 0.80-1.19 | 2 |
|  | ≤0.79 | 3 |
| TC(mmol/L) | ≥10.00 | 0 |
|  | 7.78-9.99 | 1 |
|  | 5.56-7.77 | 2 |
|  | ≤5.55 | 3 |

ALB, serum albumin concentration; TLC, total peripheral lymphocyte; TC, serum total cholesterol.

Supplement Table 2.Variance inflation factor among variables included in a multivariate logistic regression

|  | Sex | Age | ALB | Fe | TRF | CONUT |
| --- | --- | --- | --- | --- | --- | --- |
| Sex |  | 1.01 | 1 | 1.03 | 1.04 | 1.09 |
| Age |  |  | 1.14 | 1.06 | 1.09 | 1.09 |
| ALB |  |  |  | 1.08 | 1.16 | 1.44 |
| Fe |  |  |  |  | 1.02 | 1.02 |
| TRF |  |  |  |  |  | 1.08 |
| CONUT |  |  |  |  |  |  |

CONUT, controlling nutritional status score; ALB, serum albumin concentration; TRF, transferrin; Fe, iron.


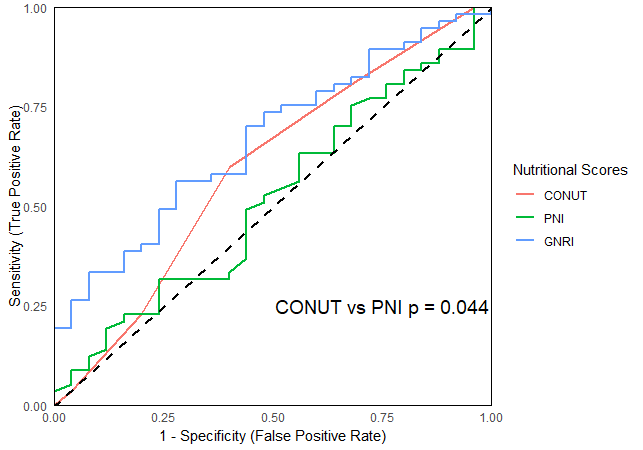


Supplement Figure 1. Receiver operating characteristic curve of three nutritional scores in individuals aged 75 years and above


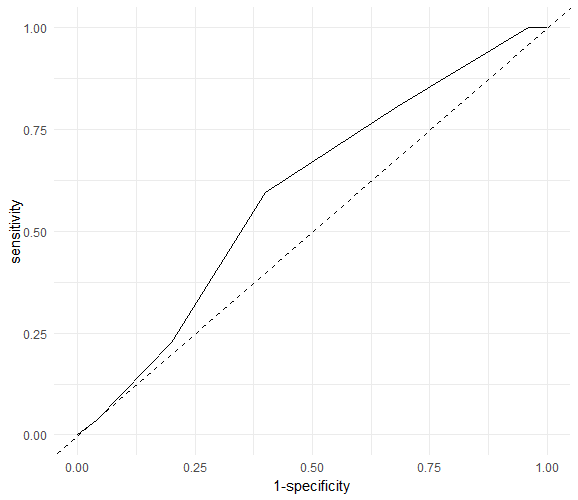


Supplement Figure 2. Receiver operating characteristic curve of CONUT score


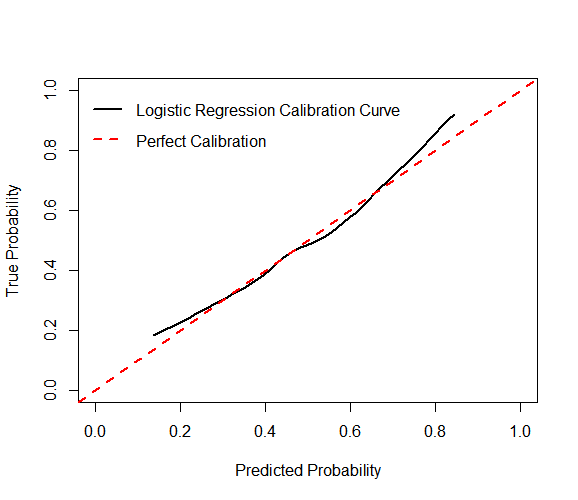


Supplement Figure 3. Calibration plots of the logistics model for sarcopenia


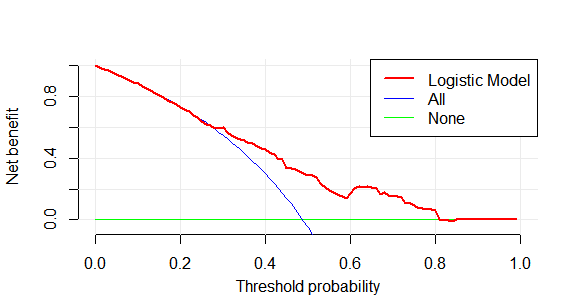


Supplement Figure 4. Decision curve analysis (DCA) of the logistics model for sarcopenia

B

A


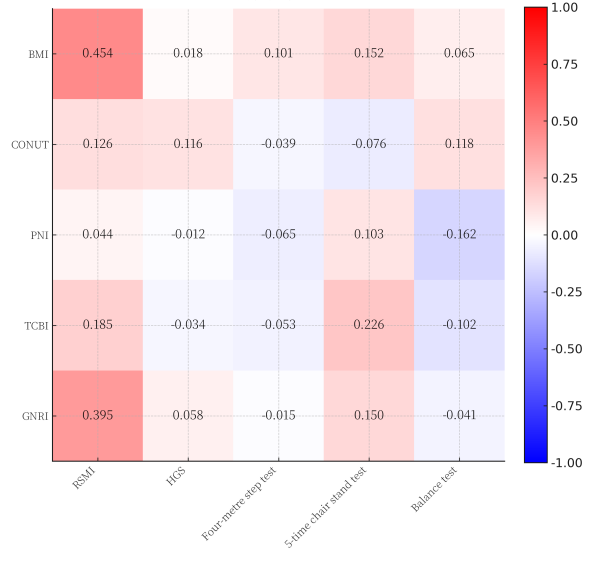

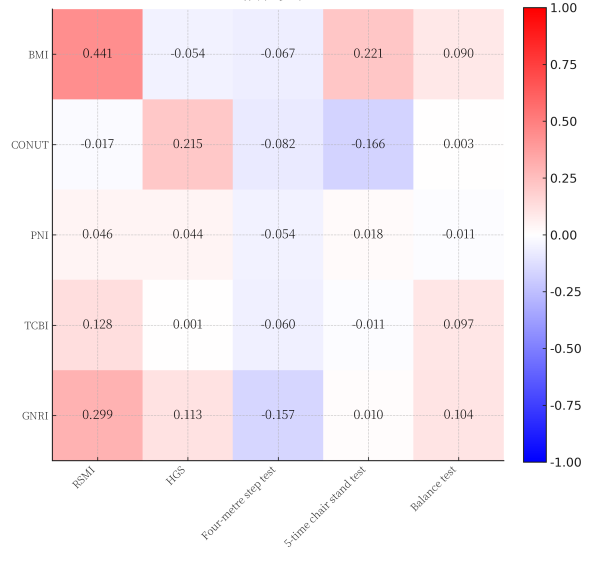


Supplement Figure 5. Heatmap of the correlations between the nutritional scores and RSMI, HGS, four-metre step test, 5-time chair stand test and balance test in patients with(A) and without sarcopenia(B).Spearman’s r was used for correlations between non-normal distribution variables and Pearson’s r for correlations between normal distribution variables, and p < 0.05 was considered statistically significant. RSMI, relative skeletal muscle mass index; HGS, handgrip strength; BMI, body mass index; CONUT, controlling nutritional status score; PNI, prognostic nutritional index; TCBI, triglyceride–total cholesterol–body weight index; GNRI, geriatric nutritional risk index.
